# Impact of dexamethasone on the incidence of ventilator-associated pneumonia in mechanically ventilated COVID-19 patients: a propensity-matched cohort study

**DOI:** 10.1186/s13054-022-04049-2

**Published:** 2022-06-13

**Authors:** Vittorio Scaravilli, Amedeo Guzzardella, Fabiana Madotto, Virginia Beltrama, Antonio Muscatello, Giacomo Bellani, Gianpaola Monti, Massimiliano Greco, Antonio Pesenti, Alessandra Bandera, Giacomo Grasselli

**Affiliations:** 1 Dipartimento di Anestesia, Rianimazione ed Emegenza Urgenza, Fondazione IRCCS Ca’ Granda Ospedale Maggiore Policlinico, Via Francesco Sforza 35, 20122 Milan, Italy; 2 Department of Biomedical, Surgical and Dental Sciences, University of Milan, Milan, Italy; 3 Department of Pathophysiology and Transplantation, University of Milan, Milan, Italy; 4 IRCCS Multimedica, Value-Based Health Care Unit, Sesto San Giovanni, Milan, Italy; 5 Infectious Diseases Unit, Fondazione IRCCS Ca’ Granda Ospedale Maggiore Policlinico, Milan, Italy; 6 Department of Anesthesia and Intensive Care Medicine, San Gerardo Hospital ASST Monza, Monza, Italy; 7 School of Medicine and Surgery, University of Milano-Bicocca, Monza, Italy; 8 Dipartimento di Anestesia e Rianimazione, ASST Grande Ospedale Metropolitano Niguarda, Milan, Italy; 9 Department of Anaesthesia and Intensive Care, Humanitas Clinical and Research Center-IRCCS, Rozzano, MI Italy; 10 Department of Biomedical Sciences, Humanitas University, Pieve Emanuele, MI Italy

**Keywords:** COVID-19, Ventilator-associated pneumonia, Intensive care unit, Hospital-acquired infections, Corticosteroids, Critical care

## Abstract

**Objective:**

To assess the impact of treatment with steroids on the incidence and outcome of ventilator-associated pneumonia (VAP) in mechanically ventilated COVID-19 patients.

**Design:**

Propensity-matched retrospective cohort study from February 24 to December 31, 2020, in 4 dedicated COVID-19 Intensive Care Units (ICU) in Lombardy (Italy).

**Patients:**

Adult consecutive mechanically ventilated COVID-19 patients were subdivided into two groups: (1) treated with low-dose corticosteroids (dexamethasone 6 mg/day intravenous for 10 days) (DEXA+); (2) not treated with corticosteroids (DEXA−). A propensity score matching procedure (1:1 ratio) identified patients' cohorts based on: age, weight, PEEP Level, PaO_2_/FiO_2_ ratio, non-respiratory Sequential Organ Failure Assessment (SOFA) score, Charlson Comorbidity Index (CCI), C reactive protein plasma concentration at admission, sex and admission hospital (exact matching).

**Intervention:**

Dexamethasone 6 mg/day intravenous for 10 days from hospital admission.

**Measurements and main results:**

Seven hundred and thirty-nine patients were included, and the propensity-score matching identified two groups of 158 subjects each. Eighty-nine (56%) DEXA+ versus 55 (34%) DEXA− patients developed a VAP (RR 1.61 (1.26–2.098), *p* = 0.0001), after similar time from hospitalization, ICU admission and intubation. DEXA+ patients had higher crude VAP incidence rate (49.58 (49.26–49.91) vs. 31.65 (31.38–31.91)VAP*1000/pd), (IRR 1.57 (1.55–1.58), *p* < 0.0001) and risk for VAP (HR 1.81 (1.31–2.50), *p* = 0.0003), with longer ICU LOS and invasive mechanical ventilation but similar mortality (RR 1.17 (0.85–1.63), *p* = 0.3332). VAPs were similarly due to G+ bacteria (mostly Staphylococcus aureus) and G− bacteria (mostly Enterobacterales). Forty-one (28%) VAPs were due to multi-drug resistant bacteria. VAP was associated with almost doubled ICU and hospital LOS and invasive mechanical ventilation, and increased mortality (RR 1.64 [1.02–2.65], *p* = 0.040) with no differences among patients' groups.

**Conclusions:**

Critically ill COVID-19 patients are at high risk for VAP, frequently caused by multidrug-resistant bacteria, and the risk is increased by corticosteroid treatment.

*Trial registration*: NCT04388670, retrospectively registered May 14, 2020.

**Supplementary Information:**

The online version contains supplementary material available at 10.1186/s13054-022-04049-2.

## Introduction

Acute respiratory distress syndrome (ARDS) is a common complication of COVID-19 that frequently requires prolonged invasive mechanical ventilation [1].

Since some randomized controlled trials [2–6] demonstrated a mortality benefit from low-dose corticosteroid therapy, early steroids treatment has become standard in severe COVID-19 [7].

A high incidence of secondary infections, particularly ventilator-associated pneumonia (VAP), has been reported in critically ill COVID-19 patients. Still, the role of corticosteroids in the risk of infectious complications remains uncertain.

This multicenter propensity-matched retrospective cohort study aims to assess the impact of treatment with steroids on the incidence and outcome of VAP in mechanically ventilated COVID-19 patients.

## Materials and methods

The local Ethical Committee approved the study (Comitato Etico Milano Area 2; #0008489).

This retrospective propensity-matched retrospective cohort was conducted in four dedicated COVID-19 ICUs in Lombardy (Italy) from February 24 to December 31, 2020. Patients' clinical management was shared between the centers (see Additional file 1).

All patients admitted to ICU with laboratory-confirmed SARS-CoV-2 infection (positive Reverse-Transcription-Polymerase Chain Reaction assay of nasal swabs) were eligible for inclusion. Exclusion criteria were: (1) age < 18 years old; (2) ICU length of stay (LOS) < 48 h; (3) respiratory co-infections at ICU admission; (4) reason for ICU admission other than COVID-19; (5) treatment with immunosuppressors (i.e., tocilizumab, anakinra) and high-dose corticosteroids (> 1 mg/kg/die methylprednisolone).

After collection of clinical variables at admission, the patients' population was subdivided into two groups: (1) patients admitted before the publication of the RECOVERY trial, who did not receive corticosteroids (DEXA−); (2) patients admitted after June 2020, who received low-dose corticosteroids as per RECOVERY protocol (dexamethasone 6 mg/day intravenous 10 days) (DEXA+). A propensity score matching procedure (1:1 ratio, caliper of 0.2) was applied to identify two cohorts of patients matched based on the following covariates: age, weight, PEEP Level, PaO_2_/FiO_2_, non-respiratory Sequential Organ Failure Assessment (SOFA) score, Charlson Comorbidity Index (CCI), C-reactive protein concentration at ICU admission, and sex and admission hospital.

The primary outcome measure was the incidence rate and etiology of microbiologically confirmed bacterial VAP (see Additional file 1). For every VAP episode, the presence of sepsis or septic shock was recorded. Multidrug-resistant (MDR) VAP was defined according to the international guidelines [8]. The following secondary outcomes were recorded: survival at ICU and hospital discharge, ICU length of stay (LOS), and duration of mechanical ventilation.

### Statistical analyses

Continuous variables were reported using median and interquartile range (IQR), while discrete variables with absolute and relative frequency.

Differences between groups were assessed using the chi-square test (or Fisher exact tests) and Student's t test (or Wilcoxon rank-sum test) as appropriate. The crude VAP incidence rate (IR) per 1000 patient-days (pd) in ICU and relative 95% confidence interval (CI) was estimated, considering only the first VAP occurrence and the days at risk between intubation and VAP, death, or ICU discharge. Risk Ratio (RR), Incidence Rate Ratio (IRR), and 95% CI were estimated as association measures between treatment and VAP occurrence.

Competing risk analysis was used to estimate the cumulative incidence of VAP in the two groups, with death as a competing event. The Grey test was applied to compare the cumulative incidence functions, and hazard ratio (95% CI) was estimated using the Fine and Gray subdistribution hazard function.

All tests were two-sided, and *p* < 0.05 was chosen to indicate statistical significance. SAS (SAS, Cary, NC, USA) and R, version 3.5.2 (R foundation, Wein, Austria) were used for statistical analysis.

## Results

Between February 24, 2020, and December 31, 2020, 952 patients were admitted to the 4 participant centers ICUs; 739 met the study inclusion criteria (see Additional file 1: Fig. S1 and Table S1), and from them, the matching procedure identified a sample of 316 subjects (158 in each group) (see Additional file 1: Table S2). Patients were primarily male (78%), overweight (body mass index 28 [25–31]), with frequent cardiologic comorbidities (i.e., hypertension 47%, diabetes 16%, CCI 2 [1–3]). Patient suffered of a mostly respiratory critical illness (i.e., non-respiratory SOFA score 0[0–1], SOFA score 4 [3, 4], PaO_2_/FiO_2_ 124 [93–180] mmHg) managed with lung-protective ventilation (PEEP 10 [10–12] cmH_2_O, tidal volume/predicted body weight 6.6 [6.0–7.4] mL/kg). Pronation was frequently employed (i.e., 65% of the patients), while renal replacement therapy and extracorporeal lung support were utilized in 8% and 4% of the patients. After matching, no clinically meaningful differences in admission parameters were observed between the patients' cohorts.

**Fig. 1:**
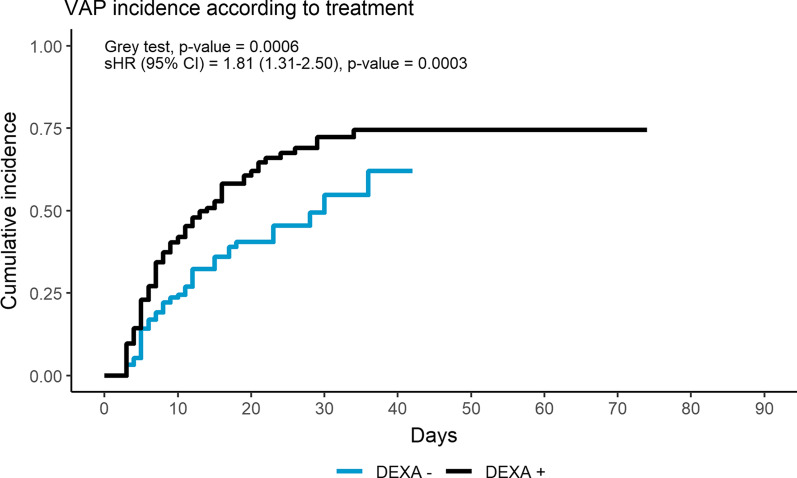
Cumulative incidence of ventilator-associated pneumonia, stratified by corticosteroids use

Eighty-nine (56%) DEXA+ patients developed a VAP, as compared to 55 (35%) DEXA− patients (RR 1.61 (1.26–2.09), *p* < 0.0001), after similar median time intervals from hospitalization, ICU admission and intubation (Table 1). The crude VAP incidence rate was higher for DEXA+ patients, 49.58 (49.26–49.91) versus 31.65 (31.38–31.91) VAP*1000/pd (IRR 1.57 (1.55–1.58), *p* < 0.0001). Competing risk analysis showed higher, statistically significant risk for VAP in the DEXA+ patients (HR 1.81 (1.31–2.50), *p* = 0.0003) (Fig. 1). DEXA+ patients showed longer ICU LOS and invasive mechanical ventilation but similar mortality (RR 1.17 (0.85–1.63), *p* = 0.3332).

**Table 1:** Patients’ outcomes

Parameter	Total (*n* = 316)	DEXA+ (*n* = 158, 50%)	DEXA (*n* = 158, 50%)	*p* value	RR/ Incidence RR (95% CI)
VAP, No. (% of the included patients)	144 (46%)	89 (56%)	55 (35%)	**0.0001**	**1.61 (1.26–2.09)**
VAP Incidence (No. × 1000 ventilation-pt/days; 95% CI)	40.76 (40.55–40.97)	49.58 (49.26–49.91)	31.65 (31.38–31.91)	**< 0 .0001**	**1.57 (1.55–1.58)**
*VAP Severity, No. (% of the included patients)*					
Infection	80 (56%)	43 (48%)	37 (67%)	**0.0496**	
Sepsis	32 (22%)	25 (28%)	7 (13%)		
Shock	32 (22%)	21 (24%)	11 (20%)		
*Outcome ICU, No. (% of the included patients)*					
Discharged	216 (68%)	104 (66%)	112 (71%)	0.3332	1.17 (0.85–1.63)
Death	100 (32%)	54 (34%)	46 (29%)		
ICU LOS (days)	15 [− 9 to 27]	17 [10–31]	14 [8–23]	**0.0225**	
Mechanical ventilation (days)	14 [9–27]	16 [10–30]	13 [7–22]	**0.0143**	
Time from hospitalization to infection (days)	10 [6–16]	10 [6–16]	10 [6–15]	0.9803	
Time from ICU to infection (days)	7 [5–12]	7 [5–12]	7 [5–12]	0.6407	
Time from intubation to infection (days)	7 [5–12]	7 [5–12]	7 [5–12]	0.5070	

Data are presented as absolute frequency (% of the included patients) or as median and interquartile range. RR, Risk Ratio; ICU, Intensive Care Unit; MDR, Multi-Drug Resistant; VAP, Ventilator-Associated Pneumonia; LOS, Length of Stay. Statistically significant variables are highlighted in bold

VAP was associated with increased overall mortality (i.e., 38% vs. 27%, OR 1.64 [1.02–2.65], *p* = 0.040). with almost doubled mechanical ventilation (i.e., 22 [14–42] vs. 12 [6–18] days, *p* < 0.001 in DEXA+ patients, and 23 [14–37] vs. 11 [5–15] days, *p* < 0.001 in DEXA− patients), and ICU LOS (i.e., 25 [14–37] vs. 11 [7–20] days, *p* < 0.001 in DEXA+ patients, and 24 [15–38] vs. 12 [6–16] days, *p* < 0.001, in DEXA− patients), with no differences among patients' groups (see Additional file 1: Tables S3, S4 and S5).

The etiology of VAP was not different between groups (see Additional file 1: Table S6). VAPs were due to G+ bacteria (mostly Staphylococcus aureus, i.e., 83% of the G+ VAPs) and G− bacteria (mostly Enterobacterales, i.e., 45% of G- VAPs) in 33% and 67% of the cases. Forty-one (28%) VAPs were due to MDR microorganisms: 26 (29%) in DEXA+ patients, and 15 (27%) (*p* = 0.802). Considering patients with VAP, DEXA+ had a higher incidence rate of MDR VAP compare to DEXA− (31.48 vs. 27.83 VAP*1000/pd), with an IRR equal to 1.13 (95% CI: 1.11–1.15; *p* < 0.0001). No significant difference between groups was detected at competing risk analysis (sHR 1.09, 95% CI: 0.58–2.04, *p* = 0.800) (see Additional file 1: Fig. S2).

## Discussion

In this study, we documented a high risk of VAP in mechanically ventilated COVID-19 patients, which was further increased by using corticosteroids.

We previously documented [1] that critically ill COVID-19 patients are at high risk for hospital-acquired infections, especially VAP and bloodstream infections, frequently caused by MDR bacteria. Several studies [9] showed similar results, with an apparent increased risk of infection in COVID-19 vs. non-COVID-19 patients [10].

Since the publication of the RECOVERY and subsequent randomized controlled studies [2–6], corticosteroids were introduced as standard clinical practice for severe COVID-19 patients. Only the CoDEX trial specifically assessed the incidence of infections [6]. However, the study was terminated early after the dissemination of the results of the RECOVERY study, and the impact of corticosteroids on infection risk could not be evaluated. A retrospective analysis [11] on the effect of corticosteroid treatment in invasively ventilated COVID-19 patients failed to show an increased risk of infections. Still, this study did not focus on VAP and included a limited number of non-matched patients (i.e., 151). Moreover, 30% of the cases received concomitant treatment with other immunosuppressants (e.g., tocilizumab), and 10% of the subjects had a secondary co-infection at admission.

To control the effect of potential confounders and of eventual selection bias in the use of steroids, in the present study we: (1) excluded all patients treated with rescue immunosuppressants (i.e., high-dose corticosteroids, tocilizumab); (2) excluded all patients with co-infections; (3) performed a rigorous matching approach—comprising the basal inflammatory status by the CRP at admission—that allowed to compare two "identical" groups of patients except for their exposure status; (4) focused only on microbiologically confirmed VAP; (5) performed a complete follow-up, until death or hospital discharge (and thus excluding the possibility for a late protective effect of steroids on VAP risk).

Our study has several limitations. First, it is a retrospective analysis. Second, there was no standard antibiotic strategy across different centers and periods. Third, since we included only microbiologically confirmed VAP, we may have underestimated the incidence of low-yield cultures (e.g., cultures obtained while the patients were receiving an antibiotic treatment) infections. Fourth, the study was conducted in a single Western European country with a high incidence of MDR infection, limiting the generalizability of our findings. Lastly, unmeasurable, and unavailable confounders (e.g., evolutionary patterns of microbiological epidemiology, different period of enrollment) may have influenced our results.

## Conclusions

Critically ill COVID-19 patients are at high risk for VAP, frequently caused by multidrug-resistant bacteria, and the risk is increased by corticosteroid treatment. Clinicians should make every effort to implement protocols for the surveillance and prevention of infectious complications. Further longitudinal studies could focus on benefits and costs of DEXA connected to VAP incidence and survival.

## Supplementary Information

**Additional file 1.** Additional methods and results.

## Data Availability

The datasets used and/or analyzed during the current study are available from the corresponding author on reasonable request.

## Abbreviations

ARDS: Acute respiratory distress syndrome
CCI: Charlson Comorbidity Index
CI: Confidence interval
COVID-19: Coronavirus disease 2019
DEXA: Dexamethasone
FiO_2_: Inspired fraction of oxygen
HR: Hazard ratio
ICU: Intensive care unit
IQR: Interquartile range
IR: Incidence rate
IRR: Incidence rate ratio
LOS: Length of stay
MDR: Multidrug-resistant
OR: Odds ratio
PaO_2_: Partial pressure of oxygen
PEEP: Positive end-expiratory pressure
RR: Risk ratio
SOFA: Sequential organ failure assessment
VAP: Ventilator-associated pneumonia
