# Supplementary material for: Impact of dexamethasone on the incidence of ventilator-associated pneumonia in mechanically ventilated COVID-19 patients: a propensity-matched cohort study
Source: Crit Care. 2022 Jun 13;26:176. doi: 10.1186/s13054-022-04049-2 (PMC9191402; doi:10.1186/s13054-022-04049-2)
Supplement: Supplementary file 1 — Additional file 1. Additional methods and results. [file 13054_2022_4049_MOESM1_ESM.docx]

**SUPPLEMENTARY MATERIAL**

**List of documents:**

1. Supplementary Methods
2. Figure S1. Patients Flowchart.
3. Table S1. Patients’ characteristics before matching.
4. Table S2. Patients’ characteristics after matching.
5. Figure S2. Cumulative incidence of VAP due to multi-drug resistant bacteria.
6. Table S3. ICU survival.
7. Table S4. Length of mechanical ventilation.
8. Table S5. ICU length of stay.
9. Table S6. VAP Etiologies.

**Supplementary Methods**

*Partecipating Centers*

This work is a retrospective analysis of prospectively collected data of all consecutive COVID-19 patients admitted to the ICUs of 4 Italian hub hospital:

- Fondazione IRCCS Ca’ Granda - Ospedale Maggiore Policlinico, Milan;

- IRCCS Istituto Clinico Humanitas, Milan;

- ASST Grande Ospedale Metropolitano Niguarda, Milan;

- Azienda Ospedaliera S. Gerardo, Monza.

*Patient clinical management*

Following HAIs management approaches were shared between participating centers: 1) routine antibiotic prophylaxis was not recommended; 2) stress ulcer and deep vein thrombosis prophylaxes were provided; 3) ventilator-associated pneumonia (VAP) bundles were applied; 4) no selective digestive decontamination was employed.

Policy for microbiological surveillance was the same in all participating centers: routine surveillance cultures for bacterial and fungal infections (perineal and nasal swabs for multidrug-resistant (MDR) bacteria, tracheal aspirate, and urine cultures) were obtained at ICU admission and then at least once a week, while further microbiological examinations were performed in the presence of a clinical/laboratory suspicion of infection.

*VAP diagnostic criteria*

VAP was diagnosed at least 48h after intubation in presence of all following criteria: 1) positivity of one culture (cut off: ≥ 10^4^ Colony Forming Units/mL for Bronchoalveolar lavage, ≥ 10^5^ CFU/mL for Endotracheal Aspirate); 2) at least 2 of the following: fever or leukocytosis/leucopenia or purulent secretions; 3) New/progressive radiographic infiltrate; 4) worsening oxygenation.

Following organisms were not considered in VAP diagnosis: “Normal respiratory flora”, “normal oral flora”, “mixed respiratory flora”, and - unless identified from lung tissue or pleural fluid (with specimen obtained during thoracentesis or initial placement of chest tube and not from an indwelling chest tube) - Candida spp, coagulase-negative staphylococci, Enterococcus spp.

**Additional Results**

**Figure S1. Patients Flowchart.**

**
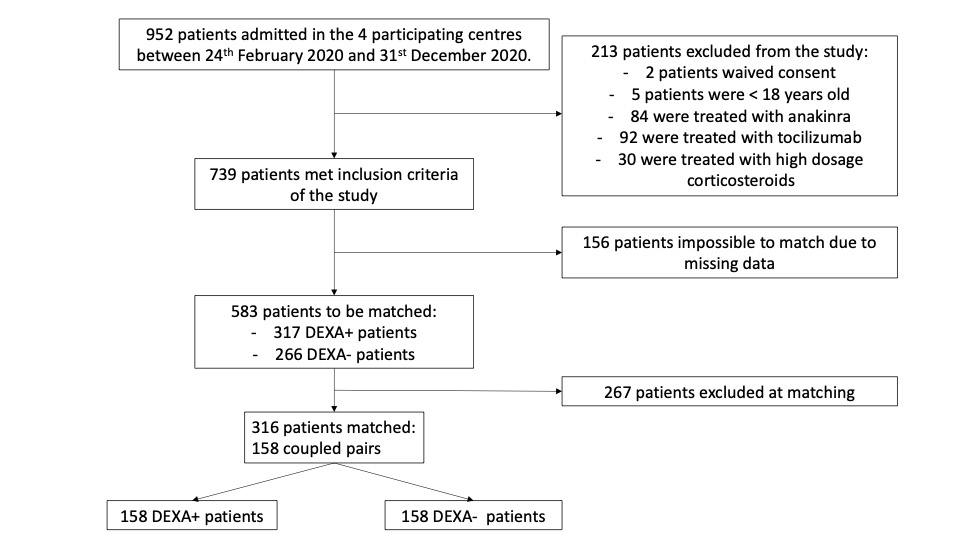
**

DEXA, Dexamethasone; ICU, Intensive Care Unit

**Table S1. Patients characteristics at ICU admission, comorbidities, and employed therapies before matching.**

|  | **Total (n = 583)** | **DEXA + (n=317, 54%)** | **DEXA - (n=266, 46%)** | **p-value** | **OR (95% CI)** |
| --- | --- | --- | --- | --- | --- |
| **Gender (female)** | 136 (23%) | 74 (23%) | 62 (23%) | 0.991 | 1.00 (0.68 – 1.47) |
| **Age (years)** | 63 [55 - 68] | 63 [55 - 68] | 63 [55 - 68] | 0.891 | 0.99 (0.98 – 1.01) |
| **BMI (kg/m2)** | 28 [25 - 31] | 29 [26 - 32] | 28 [26 - 32] | **0.016** | **1.00 (1.03– 1.07)** |
| **Charlson's Comorbidity Index** | 2 [1 - 3] | 2 [1 - 3] | 2 [1 - 3] | 0.479 | 0.97 (0.88 – 1.05) |
| **Immunologic comorbidity^1^** | 73 (13%) | 34 (11%) | 39 (15%) | 0.153 | 0.69 (0.42 – 1.14) |
| **Hypertension** | 290 (50%) | 161 (51%) | 129 (49%) | 0.581 | 1.09 (0.79 – 1.51) |
| **Diabetes** | 103 (18%) | 59 (19%) | 44 (17%) | 0.513 | 1.15 (0.75 – 1.77) |
| **SOFA Score** | 4 [3 - 5] | 4 [3 - 4] | 4 [3 - 5] | **< 0.001** | **0.75 (0.65 – 0.85)** |
| **NR-SOFA Score** | 1 [0 - 2] | 0 [0 - 1] | 0 [0 - 1] | **< 0.001** | **0.64 (0.54 – 0.76)** |
| **SAPS II Score** | 36 [32 - 42] | 35 [30 - 42] | 35 [30 - 42] | **< 0.001** | **0.95 (0.94 – 0.97)** |
| **APACHE Score** | 10 [8 - 13] | 10 [8 - 13] | 10 [8 - 13] | 0.179 | 0.97 (0.93 – 1.01) |
| **PaO_2_/FiO_2_ (mmHg)** | 123 [93 - 175] | 122 [92 - 168] | 122 [92 - 170] | 0.486 | 0.99 (0.98 – 1.01) |
| **Respiratory Rate (bpm)** | 22 [19 - 28] | 20 [18 - 25] | 20 [18 - 26] | **0.003** | **0.96 (0.94 – 0.98)** |
| **TV/PBW (mL/kg)** | 6.5 [6 - 7.4] | 6.8 [6.3 - 7.5] | 6.8 [6.1 - 7.4] | 0.136 | 1.15 (0.95 – 1.39) |
| **PEEP (cmH_2_O)** | 10 [10 - 14] | 10 [10 - 12] | 10 [10 - 13] | **< 0.001** | **0.88 (0.82 – 0.85)** |
| **Plateau Pressure (cmH_2_O)** | 23 [21 - 26] | 23 [21 - 25] | 23 [21 - 25] | 0.664 | 0.98 (0.94 – 1.04) |
| **pH** | 7.4 [7.34 - 7.46] | 7.41 [7.34 - 7.45] | 7.41 [7.34 - 7.45] | 0.712 | 1.44 (0.20 – 10.38) |
| **PaCO_2_ (mmHg)** | 43 [36 - 50] | 42 [36 - 49] | 43 [36 - 50] | 0.861 | 0.99 (0.98 – 1.01) |
| **White Blood Cells** | 8.87 [6.62 - 12.33] | 9.77 [7.26 - 13.05] | 9.45 [6.98 - 12.78] | 0.340 | 1.00 (0.99 – 1.02) |
| **Neutrophilis (10^3/mm3)** | 7.42 [5.3 - 10.92] | 8.56 [6.43 - 11.41] | 8.06 [6 - 11.1] | **0.012** | **1.04 (1.00 – 1.09)** |
| **Lymphocytes (10^3/mm3)** | 0.7 [0.5 - 1] | 0.64 [0.4 - 1] | 0.67 [0.43 - 1] | 0.616 | 1.00 (0.97 – 1.03) |
| **Neutroph/Lympho Ratio** | 10.9 [6.2 - 17.7] | 11.4 [6.8 - 18.6] | 11 [6.5 - 18.1] | **0.040** | **1.01 (0.99 – 1.03)** |
| **Platelets (10^3/mm3)** | 232 [178 - 305] | 253 [195 - 317] | 242 [185 - 311] | 0.174 | 1.00 (0.99 – 1.01) |
| **Serum Bilirubin (mg/dL)** | 0.7 [0.5 - 1.2] | 0.6 [0.4 - 0.8] | 0.6 [0.4 - 1] | **< 0.001** | **0.59 (0.43 – 0.79)** |
| **INR** | 1.23 [1.13 - 1.32] | 1.15 [1.07 - 1.25] | 1.17 [1.09 - 1.29] | 0.216 | 0.70 (0.40 – 1.24) |
| **Creatinine (mg/dL)** | 0.9 [0.7 - 1.2] | 0.8 [0.7 - 1] | 0.9 [0.7 - 1.1] | **0.004** | **0.73 (0.56 – 0.95)** |
| **LDH (units/L)** | 462 [353 - 566] | 419 [318 - 544] | 439 [340 - 560] | 0.057 | 0.99 (0.98 – 1.00) |
| **D-dimer (ng/mL)** | 1300 [567 - 4594] | 1052 [599 - 5191] | 1145 [575 - 4713] | **0.015** | **1.00 (0.99 – 1.01)** |
| **C-reactive protein (mg/dL)** | 15 [8.1 - 22.8] | 10.5 [4.5 - 17] | 12.3 [5.7 - 19.3] | **< 0.001** | **0.95 (0.94 – 0.97)** |
| **Procalcitonin (ng/mL)** | 0.5 [0.2 - 1.4] | 0.3 [0.14 - 0.7] | 0.4 [0.18 - 1.1] | **0.030** | **0.95 (0.91 – 1.00)** |
| **Ferritine (ng/mL)** | 1305 [648 - 2358] | 1159 [745 - 2205] | 1176 [710 - 2302] | 0.942 | 1.00 (0.99 – 1.01) |
| **Interleukin 6 (ng/L)** | 167 [75 - 404] | 80 [15 - 145] | 97 [35 - 225] | 0.401 | 1.00 (0.99 – 1.01) |
| Pronation | 347 (60%) | 196 (62%) | 151 (57%) | 0.215 | 1.23 (0.88 – 1.71) |
| Renal Replacement Therapy | 46 (8%) | 18 (6%) | 28 (11%) | **0.030** | **0.51 (0.27 – 0.94)** |
| Extracorporeal lung support | 13 (2%) | 9 (3%) | 4 (2%) | 0.268 | 1.91 (0.58 – 6.28) |

Data are presented as absolute frequency (% of the included patients) or as median and interquartile range. BMI, Body Mass Index; SOFA, Sequential Organ Failure Assessment; NR- SOFA: Non Respiratory Sequential Organ Failure Assessment; SAPS II, Simplified Acute Physiology Score; APACHE, ; PaO_2_, arterial oxygen partial pressure; FiO_2_, inspiratory fraction of oxygen; TV, Tidal Volume; PBW, Predicted Body Weight; PEEP, positive end expiratory pressure; PaCO_2_, arterial carbon dioxide partial pressure; INR, international normalized ratio; LDH, lactate dehydrogenase. ^a^ Including chronic immunosuppressive therapies, active hematological malignancies, neoplastic diseases, autoimmune diseases.

**Table S2. Patients' characteristics at ICU admission, comorbidities, and employed therapies, after matching.**

|  | **Total (n = 316)** | **DEXA + (n=158, 50%)** | **DEXA - (n=158, 50%)** | **p-value** | **OR (95% CI)** |
| --- | --- | --- | --- | --- | --- |
| **Gender (female)** | 68 (22%) | 34 (22%) | 34 (22%) | - | - |
| **Age (years)** | 62 [54 - 68] | 62 [56 - 67] | 62 [55 - 68] | 0.838 | 1.00 (0.8– 1.02) |
| **BMI (kg/m2)** | 28 [25 - 31] | 28 [26 - 31] | 28 [26 - 31] | 0.341 | 1.02 (0.98 – 1.07) |
| **Charlson's Comorbidity Index** | 2 [1 - 3] | 2 [1 - 3] | 2 [1 - 3] | 0.909 | 1.00 (0.90 – 1.13) |
| **Immunologic comorbidity^1^** | 40 (13%) | 18 (11%) | 22 (14%) | 0.498 | 0.79 (0.41 – 1.55) |
| **Hypertension** | 147 (47%) | 77 (49%) | 70 (44%) | 0.430 | 1.19 (0.77 – 1.86) |
| **Diabetes** | 52 (16%) | 31 (20%) | 21 (13%) | 0.128 | 1.59 (0.57 – 2.91) |
| **SOFA Score** | 4 [3 - 4] | 4 [3 - 4] | 4 [3 - 4] | 0.368 | 1.09 (0.90 – 1.31) |
| **NR-SOFA Score** | 0 [0 - 1] | 0 [0 - 1] | 0 [0 - 1] | 0.591 | 1.06 (0.84 – 1.35) |
| **SAPS II Score** | 36 [32 - 44] | 35 [30 - 41] | 35 [30 - 43] | 0.080 | 0.98 (0.95 – 1.00) |
| **APACHE Score** | 9 [7 - 12] | 10 [8 - 13] | 10 [8 - 12] | 0.076 | 1.05 (0.99 – 1.11) |
| **PaO_2_/FiO_2_ (mmHg)** | 124 [93 - 180] | 118 [89 - 160] | 120 [91 - 170] | 0.314 | 0.99 (0.99 – 1.01) |
| **Respiratory Rate (bpm)** | 22 [18 - 28] | 20 [18 - 26] | 20 [18 - 28] | 0.432 | 0.99 (0.96 – 1.02) |
| **TV/PBW (mL/kg)** | 6.6 [6.0 - 7.4] | 6.6 [6.2 - 7.3] | 6.6 [6.1 - 7.3] | 0.738 | 0.95 (0.72 – 1.26) |
| **PEEP (cmH_2_O)** | 10 [10 - 12] | 10 [10 - 12] | 10 [10 - 12] | 0.661 | 0.97 (0.89 – 1.07) |
| **Plateau Pressure (cmH_2_O)** | 23 [20 - 26] | 24 [22 - 26] | 23 [21 - 26] | **0.012** | **1.12 (1.02 – 1.24)** |
| **pH** | 7.41 [7.35 - 7.47] | 7.41 [7.34 - 7.45] | 7.41 [7.35 - 7.46] | 0.097 | 0.09 (0.01 – 1.56) |
| **PaCO_2_ (mmHg)** | 41 [35 - 48] | 42 [36 - 49] | 41 [36 - 48] | 0.088 | 1.02 (0.99 – 1.04) |
| **White Blood Cells** | 8.61 [6.1 - 11.74] | 9.79 [7.13 - 13.08] | 9.26 [6.75 - 12.3] | 0.086 | 1.01 (0.99 – 1.05) |
| **Neutrophilis (10^3/mm3)** | 7.23 [4.91 - 10.4] | 8.66 [6.38 - 11.44] | 7.9 [5.79 - 10.83] | **0.018** | **1.05 (1.00 – 1.12)** |
| **Lymphocytes (10^3/mm3)** | 0.75 [0.53 - 0.95] | 0.65 [0.46 - 0.97] | 0.7 [0.49 - 0.97] | 0.442 | 1.01 (0.98 – 1.04) |
| **Neutroph/Lympho Ratio** | 10.1 [6 - 16.4] | 12.3 [7.9 - 22.6] | 11 [7 - 18.1] | **0.002** | **1.04 (1.01 – 1.07)** |
| **Platelets (10^3/mm3)** | 236 [182 - 306] | 250 [195 - 319] | 243 [188 - 312] | 0.436 | 1.00 (0.99 – 1.01) |
| **Serum Bilirubin (mg/dL)** | 0.7 [0.5 - 1] | 0.6 [0.4 - 0.9] | 0.6 [0.5 - 0.9] | 0.139 | 0.77 (0.54 – 1.10) |
| **INR** | 1.23 [1.14 - 1.32] | 1.16 [1.08 - 1.26] | 1.19 [1.1 - 1.29] | 0.113 | 0.46 (0.16 – 1.34) |
| **Creatinine (mg/dL)** | 0.9 [0.7 - 1.1] | 0.8 [0.7 - 1.1] | 0.9 [0.7 - 1.1] | 0.650 | 1.09 (0.75 – 1.57) |
| **LDH (units/L)** | 461 [353 - 556] | 461 [347 - 571] | 461 [352 - 563] | 0.968 | 0.99 (0.98 – 1.00) |
| **D-dimer (ng/mL)** | 1091 [502 - 4065] | 1089 [512 - 5977] | 1090 [503 - 4632] | 0.103 | 0.99 (0.98 – 1.01) |
| **C-reactive protein (mg/dL)** | 12.7 [7.1 - 18.7] | 12.4 [6.7 - 19.2] | 12.6 [6.8 - 18.9] | 0.853 | 0.99 (0.97 – 1.02) |
| **Procalcitonin (ng/mL)** | 0.4 [0.2 - 1.12] | 0.34 [0.15 - 0.83] | 0.4 [0.18 - 1.02] | 0.133 | 0.96 (0.90 – 1.01) |
| **Ferritine (ng/mL)** | 1269 [723 - 1751] | 1203 [659 - 1968] | 1212 [723 - 1778] | 0.685 | 1.00 (0.99 – 1.01) |
| **Interleukin 6 (ng/L)** | 113 [25 - 320] | 29 [14 - 96] | 52 [17 - 153] | 0.150 | 1.00 (0.99 – 1.01) |
| **Pronation** | 206 (65%) | 112 (70%) | 94 (60%) | **0.033** | **1.66 (1.04 – 2.65)** |
| **Renal Replacement Therapy** | 24 (8%) | 8 (5%) | 16 (10%) | 0.086 | 0.47 (0.20 – 1.14) |
| **Extracorporeal lung support** | 13 (4%) | 7 (4%) | 4 (3%) | 0.354 | 1.78 (0.51 – 6.22) |

Data are presented as absolute frequency (% of the included patients) or as median and interquartile range. OR, Odds Ratio; BMI, Body Mass Index; SOFA, Sequential Organ Failure Assessment; NR- SOFA: Non Respiratory Sequential Organ Failure Assessment; SAPS II, Simplified Acute Physiology Score; APACHE, ; PaO_2_, arterial oxygen partial pressure; FiO_2_, inspiratory fraction of oxygen; TV, Tidal Volume; PBW, Predicted Body Weight; PEEP, positive end expiratory pressure; PaCO_2_, arterial carbon dioxide partial pressure; INR, international normalized ratio; LDH, lactate dehydrogenase. ^a^ Including chronic immunosuppressive therapies, active hematological malignancies, neoplastic diseases, autoimmune diseases.

**Figure S2. Cumulative incidence of VAP due to multi-drug resistant bacteria in the study population, stratified by corticosteroids use.
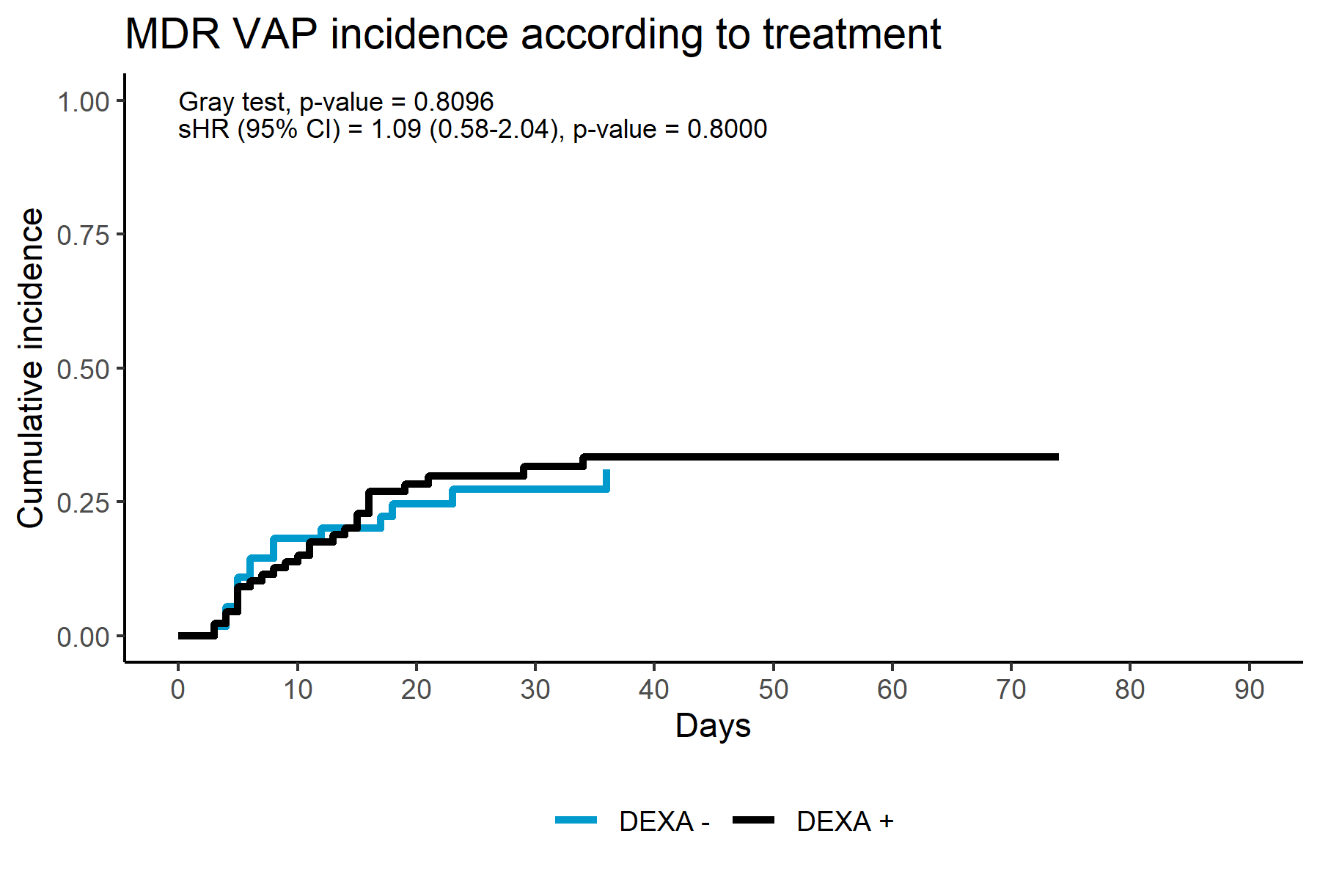
**

**Table S3. ICU survival**

|  | **DEXA + (n=158)** | | **DEXA – (n=158)** | | **Overall** | | **OR (95% CI)** | **p-value** |
| --- | --- | --- | --- | --- | --- | --- | --- | --- |
|  | N. | N. Death | N. | N. Death | N. | N. Death |  |  |
| **VAP (n=144)** | 89 | 35 (39%) | 55 | 19 (35%) | 144 | 54 (38%) | 1.23 [0.61 – 2.47] | 0.564 |
| **NO VAP (n=172)** | 69 | 19 (28%) | 103 | 27 (26%) | 172 | 46 (27%) | 1.07 [0.54 – 2.13] | 0.847 |
| **Overall** | 158 | 54 (34%) | 158 | 46 (29%) | 316 | 100 (32%) | 1.26 [0.79 – 2.03] | 0.333 |
| **OR (95% CI)** | 1.71 [0.87 – 3.36] | | 1.49 [0.73 – 3.02] | | 1.64 [1.02 – 2.65] | | - | - |
| **p-value** | 0.119 | | 0.275 | | 0.040 | | - | - |

DEXA, Dexamethasone; VAP, Ventilator Associated Pneumonia; OR, Odds Ratio.

**Table S4. Lenght of mechanical ventilation**

|  | **DEXA +** | | **DEXA –** | | **Overall** | | **p-value** |
| --- | --- | --- | --- | --- | --- | --- | --- |
|  | N. | Intubation (days) | N. | Intubation (days) | N. | Intubation (days) |  |
| **VAP** | 89 | 22 [14 – 42] | 55 | 23 [14 – 37] | 144 | 25 [14 – 40] | 0.783 |
| **NO VAP** | 69 | 12 [6– 18] | 103 | 11 [5 – 15] | 172 | 11 [6 – 16] | 0.103 |
| **Overall** | 158 | 16 [10 – 32] | 158 | 13 [7– 22] | 316 | 14 [9 – 27] | - |
| **p-value** | < 0.001 | | < 0.001 | | - | | - |

DEXA, Dexamethasone; VAP, Ventilator Associated Pneumonia.

**Table S5. ICU lenght of stay.**

|  | **DEXA +** | | **DEXA –** | | **Overall** | | **p-value** |
| --- | --- | --- | --- | --- | --- | --- | --- |
|  | N. | ICU LOS (days) | N. | ICU LOS (days) | N. | ICU LOS (days) |  |
| **VAP** | 89 | 25 [14 – 37] | 55 | 24 [15 – 38] | 144 | 24 [15 – 38] | 0.416 |
| **NO VAP** | 69 | 11 [7 – 20] | 103 | 12 [6 – 16] | 172 | 11 [6 – 18] | 0.140 |
| **Overall** | 158 | 17 [10 – 31] | 158 | 14 [8 – 23] | 316 | 15 [9 – 27] | - |
| **p-value** | < 0.001 | | < 0.001 | | - | | - |

DEXA, Dexamethasone; VAP, Ventilator Associated Pneumonia; ICU, Intensive Care Unit; LOS, Length of Stay.

**Table S6. VAP Etiologies.**

|  |  | Overall **(n=144)** | | DEXA + **(n=89)** | DEXA **– (n=55)** | **MDR (n=41)** |
| --- | --- | --- | --- | --- | --- | --- |
| **Gram Staining** | **Microorganism** |  | |  |  |  |
| G + |  | 48 (33%) | 25 (28%) | | 23 (42%) | 11 (27%) |
|  | Staphylococcus aureus | 40 (83%) | 21 (84%) | | 19 (83%) | 11 (100%) |
|  | Enterococcus spp | 4 (8%) | 1 (4%) | | 3 (13%) | - |
|  | Streptococcus Pneumoniae | - | - | | - | - |
|  | Other | 4 (9%) | 3 (12%) | | 1 (4%) | - |
| G - |  | 96 (67%) | 64 (62%) | | 32 (58%) | 30 (73%) |
|  | P. aeruginosa | 36 (38%) | 24 (38%) | | 12 (38%) | 12 (40%) |
|  | Enterobacterales (other) | 19 (20%) | 9 (14%) | | 10 (31%) | 6 (20%) |
|  | Klebsiella spp | 17 (18%) | 15 (23%) | | 2 (6%) | 1 (3%) |
|  | E. Coli | 7 (7%) | 4 (7%) | | 3 (9%) | 2 (7%) |
|  | A. baumannii | 6 (6%) | 6 (9%) | | - | 6 (20%) |
|  | Other | 11 (11%) | 6 (9%) | | 5 (16%) | 3 (10%) |

**MDR, multi-drug resistant.**
